# Associations between retinal morphological features and risk of depression and anxiety disorders

**DOI:** 10.1186/s12916-026-04969-8

**Published:** 2026-06-05

**Authors:** Yi Li, Yuzhou Zhang, Charlene Yim, Ka Wai Kam, Mary Ho, Xiu Juan Zhang, Patrick Ip, Mandy P. H. Ng, Alvin L. Young, Clement C. Tham, Chi Pui Pang, Li Jia Chen, Jason C. Yam

**Affiliations:** 1 Department of Ophthalmology and Visual Sciences, The Chinese University of Hong Kong, Hong Kong SAR, China; 2 Hong Kong Eye Hospital, Hong Kong SAR, China; 3 Department of Ophthalmology, Hong Kong Children’s Hospital, Hong Kong SAR, China; 4 Department of Ophthalmology and Visual Sciences, Prince of Wales Hospital, Hong Kong SAR, China; 5 Department of Ophthalmology, The University of Hong Kong, Hong Kong SAR, China; 6 Department of Paediatrics and Adolescent Medicine, Li Ka Shing Faculty of Medicine, The University of Hong Kong, Hong Kong SAR, China; 7 Hong Kong Hub of Paediatric Excellence, The Chinese University of Hong Kong, Hong Kong SAR, China; 8 Shantou University, The Chinese University of Hong Kong Joint Shantou International Eye Centre, Shantou, China

**Keywords:** Retinal features, Depression, Anxiety, Optical coherence tomography

## Abstract

**Background:**

Understanding how retinal morphological features are associated with the risk of developing depression and anxiety disorders holds significant implications for public health and early detection strategies. This study aims to investigate the association between optical coherence tomography (OCT)-detected retinal features and the incidence of depression and anxiety.

**Methods:**

This cohort study was conducted using data from UK Biobank participants aged 40–70 years who underwent retinal OCT imaging at recruitment. Baseline retinal morphological features, including the retinal nerve fibre layer (RNFL), ganglion cell-inner plexiform layer (GCIPL), inner nuclear layer (INL), photoreceptor layer (PRL), retinal pigment epithelium (RPE), and macular thickness, were segmented from macular-centered OCT images. Depression and anxiety disorders were identified using International Classification of Diseases (ICD) codes. Cox proportional hazards regression models were used to assess the association of retinal features with incident depression and anxiety, with the full model adjusting for demographic, lifestyle, and comorbid factors.

**Results:**

A total of 36,220 participants were included (mean [SD] age, 55.90 [8.22] years, 47.8% male). Over a median follow-up duration of 12.5 years, 1340 new cases of depression and 1373 new cases of anxiety were observed. In fully adjusted Cox hazard regression models, each standard deviation (SD) increase in GCIPL and macular thickness was associated with a lower risk of incident depression (HR [95%CI], 0.92 [0.87, 0.97]; and 0.91 [0.86, 0.96], respectively). The associations of GCIPL and macular thickness with incident depression were more pronounced among females. On the other hand, there was no association between retinal features and incident anxiety disorders in the fully adjusted model.

**Conclusions:**

Thinner GCIPL and macular thickness were independently associated with increased risk of depression, especially in females. Our findings highlight a potential role of OCT-detected retinal features as additional biomarkers for at-risk stratification of depression.

**Supplementary Information:**

The online version contains supplementary material available at 10.1186/s12916-026-04969-8.

## Background

Depression and anxiety are the two common and potentially disabling mental illnesses that significantly contribute to the global health burden [1]. According to the World Health Organization, approximately 332 million individuals worldwide are affected by depression, with women exhibiting a 1.5-fold increased likelihood of being affected compared to men. Depression not only leads to physical illnesses, impairs the quality of life, in severe cases, it also elevates the risk of suicide attempts and increases the mortality rate in patients as it is related to multiple medical comorbidities [2, 3]. Anxiety disorders, another prevalent psychological issue, are characterized as chronic illnesses that cause distress or decreased functional capabilities; the lifetime prevalence ranges from 4.8% to 31.0% across 17 countries [4, 5]. Identifying patients at risk for developing mental illnesses is a critical concern in clinical settings. Timely diagnosis is essential, and prevention plays a key role. Therefore, recognizing accessible biomarkers, particularly neuro-biomarkers, that indicate a higher risk of depression or anxiety disorders is of great importance. Currently, magnetic resonance imaging (MRI)-based studies have identified several brain structural alterations associated with these conditions, such as reduced grey matter volume, smaller cortical surface area, and compromised white matter integrity [6–9]. However, MRI has the disadvantages of high costs, time-consuming, and limited availability, which hinder its utility in facilitating the early diagnosis of mental health disorders. To tackle these challenges, there is an urgent need for objective, accessible, and accurate methods to assess risk and enable the early detection of depression and anxiety disorders.

The retina and the brain were developed from the same embryonic origin, sharing numerous anatomical and functional features, therefore the retina is considered as part of the central nervous system and remains a focus for studying the central nervous system [10, 11]. Recently, optical coherence tomography (OCT) has emerged as a non-invasive imaging tool for detailed examination of the retinal morphology in vivo, which may provide important information for the diagnosis and monitoring of neurodegeneration and mental illnesses [12, 13]. The correlations between OCT-detected retinal features with the incidence and prevalence of various neurodegenerative disorders, such as Alzheimer’s disease, Parkinson disease, and multiple sclerosis, have been established [14–16]. Moreover, case control studies have demonstrated significant retinal morphological differences in patients with schizophrenia compared to healthy control [17, 18], which suggested the potential of retinal morphological changes for the detection of psychiatric disorders. However, evidence linking retinal features to depression and anxiety disorders remains limited and constrained by cross-sectional designs [19, 20], and no prior study has looked into the relationship between OCT-detected retinal morphology and the onset and development of these conditions.

Based on the data from a nationally representative sample of UK adults, we aimed to investigate the longitudinal relationships between retinal morphology and subsequent risks of depression and anxiety disorders and identify the potential retinal features that are associated with depression and anxiety disorders in middle and older age.

## Methods

### Study participants

The baseline survey of UK Biobank was conducted from 2006 to 2010, and more than 500,000 participants aged 40 to 70 years were recruited from one of the 22 assessment centers across England, Scotland, and Wales [21]. Participants’ baseline information was collected through touchscreen questionnaires, verbal interviews, and physical measurements. A subset of 67,074 UK Biobank participants underwent OCT examinations. Of these, 53,023 were considered to have high-quality OCT images after quality control. After further excluding those with low vision (logMAR worse than 0.1), abnormal IOP measurement (more than 22 mmHg or less than 5 mmHg), diagnosed with depression and anxiety disorders at baseline, and missing demographic data, a total of 36,220 participants were included in the current analysis. The flowchart illustrating the inclusion of participants in our present study is illustrated in Figure S1 in Additional file 1.

### Retinal imaging

Macular-centered OCT imaging was performed at the initial assessment visit using TOPCON 3D OCT 1000 Mk2 (Topcon Corporation, Tokyo, Japan). This system has an axial resolution of 6 μm and an acquisition speed of 18,000 A-scans per second. All OCT images were obtained via 3-dimensional volume scans covering a 6 × 6-mm raster pattern, consisting of 128 B-sans and 512 A-scans per B scan [22]. Retinal measurements were automatically estimated using the Topcon Advanced Boundary Segmentation (TABS) tool (version 1.6.2.6), a software for automated segmentation of retinal sublayers based on dual-scale gradient [22]. Six retinal features were included: retinal nerve fibre layer (RNFL), ganglion cell-inner plexiform layer (GCIPL), inner nuclear layer (INL), photoreceptor layer (PRL), and retinal pigment epithelium (RPE), and total macular thickness. PRL was defined as the thickness between the INL and RPE in this study [23]. Several segmentation indicators were calculated beyond the layer detection processing. For image quality control, participants with image quality score less than 45 or poor segmentation certainty (poorest 20% of images based on segmentation indicators such as validity count, internal limiting membrane indicator, and minimum motion correlation) via TBAS software were excluded [24]. The average value of the data from both eyes was included for analysis, and if data from only one eye was available, the data from that eye was used.

### Ascertainment of outcomes

Diseases at baseline were defined if participants reported that they had been diagnosed with the disease by a doctor. Additional disease cases at baseline were identified through inpatient records. Incident cases of depression (F32, F33) and anxiety disorders (F40, F41) were identified by the International Classification of Diseases, 10th Revision (ICD-10) codes in medical records. At the time of analysis, hospital admission data were available for participants until 31 October 2022. Individuals were considered at risk from the assessment until the date of the first occurrence of diseases, date of death, date lost to follow-up, or end of available follow-up, whichever occurred first.

### Assessment of covariates

Covariates included age at baseline, sex, ethnicity, Townsend Deprivation Index (TDI), smoking status, alcohol drinking status, education level, body mass index (BMI), spherical equivalent (SE), intraocular pressure (IOP), hypertension, and diabetes, which were collected at baseline. TDI adopted census data on employment, housing, and social class based on the postal code of participants [25]. BMI was calculated as weight(kg)/height(m)^2^, with weight and height measured at the baseline visit. SE was calculated by sphere degree plus 0.5 multiply cylinder degree using automated refraction (RC-5000; Tomey, Japan). IOP was measured with the Ocular Response Analyzer (ORA; Reichert, USA). Hypertension was identified by self-reported diagnoses and use of antihypertensive medications. Diabetes was recorded from self-reported diagnoses and use of antidiabetic medications.

### Statistical analysis

Baseline characteristics are presented as mean (SD) for continuous variables and as number (percentage) for categorical variables. Normality tests indicated that all OCT parameters had P-values greater than 0.05 in the Kolmogorov-Smirnov test. The distribution of these parameters is illustrated in a histogram (Figure S2 in Additional file 1). To ensure comparability across variables, OCT parameters were standardized using Z-scores [15]. Multivariable-adjusted Cox proportional hazards regression models were established to assess the association between OCT parameters and the risk of depression and anxiety disorders. The associations are presented as hazard ratios (HRs) and 95% confidence intervals (CIs). Non-linear relationships between OCT parameters and risk of depression and anxiety disorders were assessed using restricted cubic spline (RCS) analyses with four knots at the 5, 35, 65 and 95 percentiles. Covariates include age, sex, TDI, ethnicity, education level, smoking status, alcohol drinking status, intraocular pressure, SE, hypertension, and diabetes. The proportional hazards assumption was tested by Schoenfeld residuals (*P* > 0.05 for all Schoenfeld global tests).

Further subgroup analyses were carried out to stratify age (≤ 60, > 60 years), sex (male, female), ethnicity (White, Non-white), education level (college or university degree, others), IOP (≤ 15, > 15 mmHg), SE (≤-0.5 diopters, >-0.5 diopters), smoking status (never, previous or current), alcohol drinking status (never, previous or current), BMI (< 25, ≥ 25), hypertension (yes, no), and diabetes (yes, no). The modification effect was tested using likelihood ratio tests, which compared models with and without a cross-product interaction term of each retinal feature and the stratified variables. Moreover, a series of sensitivity analyses were conducted to verify the robustness of our results. First, participants who experienced disease events within the first 4 years of follow-up were excluded. Second, we excluded participants with baseline self-reported ocular diseases (*n* = 2623), including cataract, glaucoma, age-related macular degeneration, diabetic retinopathy, vision loss caused by injury or trauma, and other serious eye diseases or with high refractive errors ( > ± 6 diopters, *n* = 1158) to reduce the confounding effects from ocular pathology and potential magnification artifacts on retinal measurements. Third, we excluded participants with specific retinal conditions, including glaucoma (*n* = 399), age-related macular degeneration (*n* = 288), or diabetic retinopathy (*n* = 286) separately. Fourth, participants with baseline neurological disorders (*n* = 519), including Parkinson disease, Alzheimer’s disease, and stroke were excluded. Finally, our models were additionally adjusted for physical activity, sleep duration, and the intake of mood-affecting medications (including antidepressants, antipsychotics, anxiolytics, mood stabilizers, beta-blockers, corticosteroids, and anticonvulsants) to minimize potential residual confounding. All analyses were conducted using SPSS (version 27.0; IBM Corp., Armonk, NY) and R statistical software (version 4.5.1). To account for multiple testing, we used the Benjamini–Hochberg procedure to control the false discovery rate (FDR) [26]. FDR-adjusted *P*-value < 0.05 was considered statistically significant, while FDR-adjusted *P*-value between 0.05 and 0.1 was considered nominally significant, warranting future investigation given the exploratory aspect of this study. All statistical tests were two-sided.

## Results

A total of 36,220 participants were included in the analysis. Participants had a mean (SD) age of 55.90 (8.22) years; 17,331 (47.8%) were men, and 33,148 (91.5%) were White (Table 1). Participants with eligible OCT imaging data were younger, male, White, more educated, smoked less frequently, had lower BMI, and less likely to have hypertension and diabetes than those who were excluded (all *P* < 0.05) (Additional file 1: Table S1).

**Table 1:** Baseline characteristics of participants

	Study sample (*N* = 36,220)	
		(Minimum, Maximum)
**Demographics**		
Age, mean (SD), year	55.90 (8.22)	(40, 70)
Sex, male, No. (%)	17,331 (47.8%)	
TDI, mean (SD)	-1.14 (2.92)	(-6.26, 9.16)
Ethnicity, White, No. (%)	33,148 (91.5%)	
Education level, college or university degree, No. (%)	13,587 (37.5%)	
Smoking status, No. (%)		
Never	20,290 (56.0%)	
Previous	12,652 (34.9%)	
Current	3278 (9.1%)	
Alcohol drinking status, No. (%)		
Never	1529 (4.2%)	
Previous	1116 (3.1%)	
Current	33,575 (92.7%)	
BMI, mean (SD)	27.20 (4.64)	(14.53, 65.98)
Spherical equivalent, mean (SD), D	-0.26 (2.33)	(-16.06, 9.56)
Intraocular pressure, mean (SD), mmHg	15.44 (2.95)	(5.01, 22.0)
Hypertension, No. (%)	8984 (24.8%)	
Diabetes, No. (%)	1688 (4.7%)	
**Retinal features**		
RNFL, mean (SD), micron	28.58 (4.20)	(10.91, 64.77)
GCIPL, mean (SD), micron	74.56 (5.54)	(49.05, 108.43)
INL, mean (SD), micron	32.54 (2.30)	(21.12, 45.74)
PRL, mean (SD), micron	142.20 (7.64)	(105.42, 183.27)
RPE, mean (SD), micron	25.28 (2.78)	(18.22, 77.06)
Macular thickness, mean (SD), micron	277.89 (13.19)	(202.15, 379.74)

Data were presented as mean (standard deviation) for continuous variables and No.% for categorical variables

During a median follow-up of 12.5 years (interquartile range, 12.4–12.6), 1340 incident cases of depression (3.7%) and 1373 incident cases of anxiety disorder (3.8%) were observed. Individuals with incident depression exhibited lower baseline RNFL, GCIPL, PRL, and macular thickness, while those with incident anxiety showed reduced PRL thickness (Additional file 1: Table S2). In the Cox proportional hazard analyses (Table 2), thinner RNFL, GCIPL, PRL, and macular thickness at baseline were related to incident depression in the basically adjusted model (All FDR-adjusted *P* < 0.05). In the fully adjusted models, each standard deviation (SD) increase in baseline GCIPL and macular thickness was significantly associated with a reduced risk of incident depression, with the HRs [95%CI] of 0.92 [0.87, 0.97] (*P* = 0.003) and 0.91 [0.86, 0.96] (*P* = 0.001), respectively. Although a similar inverse association was observed for PRL (HR:0.93, 95%CI: 0.88–0.99; *P* = 0.016), this finding did not remain statistically significant after FDR correction. On the other hand, a thicker INL was marginally associated with a heightened risk of incident anxiety disorders (per SD increase HR [95%CI], 1.06 [1.01, 1.12]; *P* = 0.027) in basically adjusted model, however, this association was attenuated after full adjustment for potential confounders. Other OCT parameters showed no association with incident anxiety.

**Table 2:** Association between retinal features and incident depression and anxiety disorders

	Model 1		Model 2	
	Per SD increase HR (95%CI)	*P* value	Per SD increase HR (95%CI)	*P* value
**Depression**				
RNFL	0.93 (0.88, 0.99)	0.015*	0.95 (0.90, 1.00)	0.07
GCIPL	0.93 (0.88, 0.99)	0.014*	0.92 (0.87, 0.97)	0.003*
INL	1.01 (0.95, 1.06)	0.84	0.99 (0.93, 1.05)	0.69
PRL	0.93 (0.88, 0.98)	0.006*	0.93 (0.88, 0.99)	0.016*
RPE	0.99 (0.94, 1.05)	0.70	0.99 (0.94, 1.05)	0.72
Macular thickness	0.91 (0.86, 0.96)	< 0.001*	0.91 (0.86, 0.96)	0.001*
**Anxiety**				
RNFL	0.97 (0.92, 1.02)	0.24	0.99 (0.93, 1.04)	0.67
GCIPL	1.00 (0.95, 1.06)	0.97	0.98 (0.93, 1.04)	0.53
INL	1.06 (1.01, 1.12)	0.027*	1.04 (0.98, 1.10)	0.16
PRL	0.97 (0.91, 1.02)	0.21	0.96 (0.91, 1.02)	0.20
RPE	1.02 (0.97, 1.08)	0.46	1.02 (0.96, 1.07)	0.56
Macular thickness	0.98 (0.93, 1.04)	0.48	0.97 (0.92, 1.03)	0.37

Abbreviations: RNFL, retinal nerve fiber layer; GCIPL, ganglion cell-inner plexiform layer; INL, inner nuclear layer; PRL, photoreceptor layer; RPE, retinal pigment epithelium; BMI, body mass index; TDI, Townsend deprivation index; IOP, intraocular pressure; SE, spherical equivalent

Model 1 was adjusted for age, sex, TDI, ethnicity, education level;

Model 2 was adjusted for age, sex, TDI, ethnicity, education level, smoking status, alcohol drinking status, BMI, SE, IOP, hypertension, and diabetes

* indicate FDR-corrected *P* < 0.1; Bold value indicate FDR-corrected *P* < 0.05

**Fig. 1:**
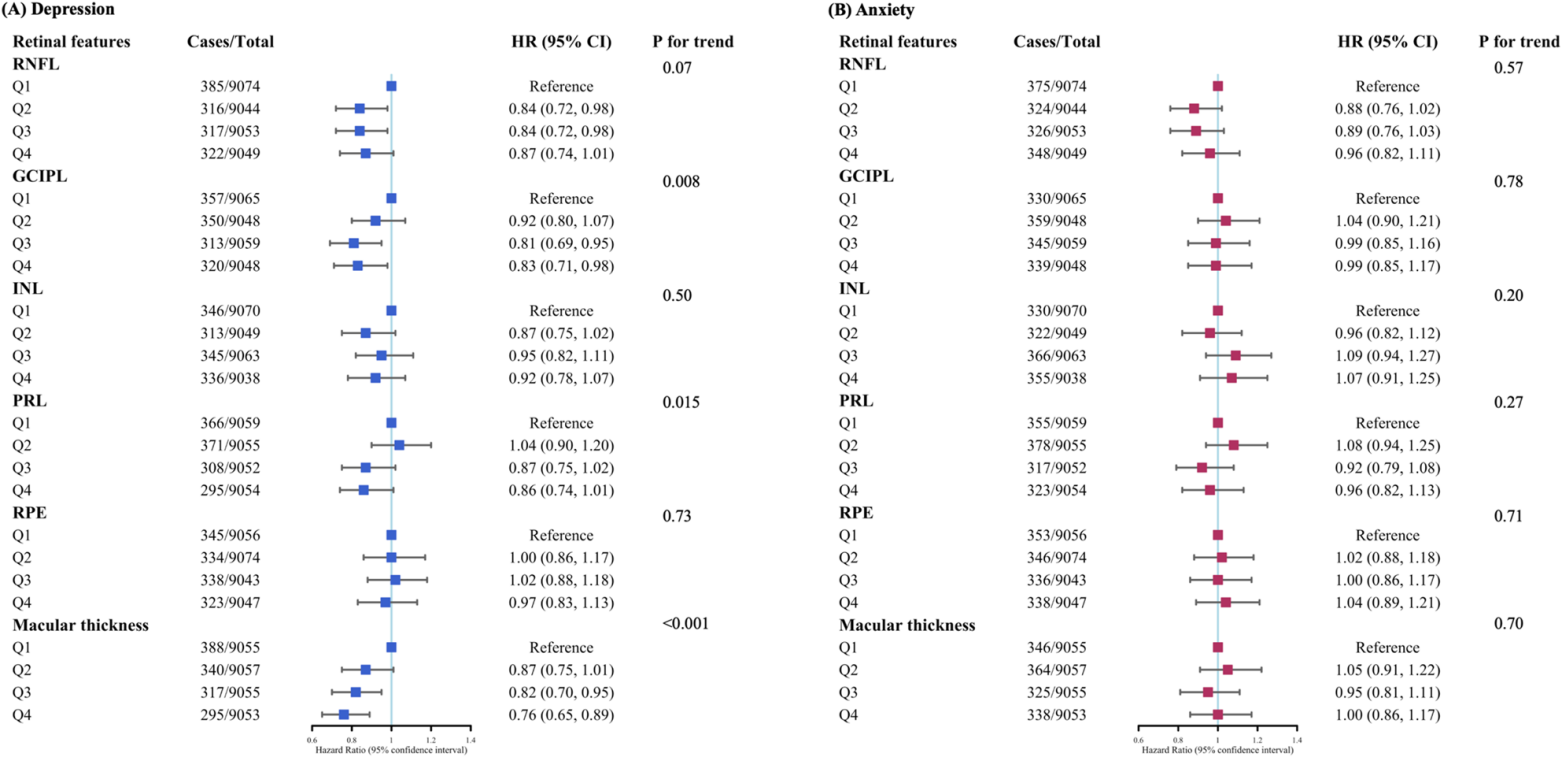
Associations between quartiles of retinal layer thickness and incident depression and anxiety disorders

Associations between quartiles of retinal layer thickness and the risk of depression and anxiety are shown in Fig. 1. Fully adjusted models revealed an inverse dose-response relationship between GCIPL, PRL, and macular thickness and depression risk (All *P* for trend < 0.05). Compared to the lowest quartile, participants in the third and fourth quartiles of GCIPL and macular thickness demonstrated a lower risk of incident depression. Specifically, individuals in the top quartile of GCIPL had a 17% reduced risk of depression (HR: 0.83, 95%CI: 0.71–0.98), while those in the top quartile of macular thickness showed a 24% reduced risk (HR: 0.76, 95%CI: 0.65–0.89), relative to their respective bottom quartiles. Conversely, no significant associations were observed between any retinal layer thickness quartiles and incident anxiety.

**Fig. 2:**
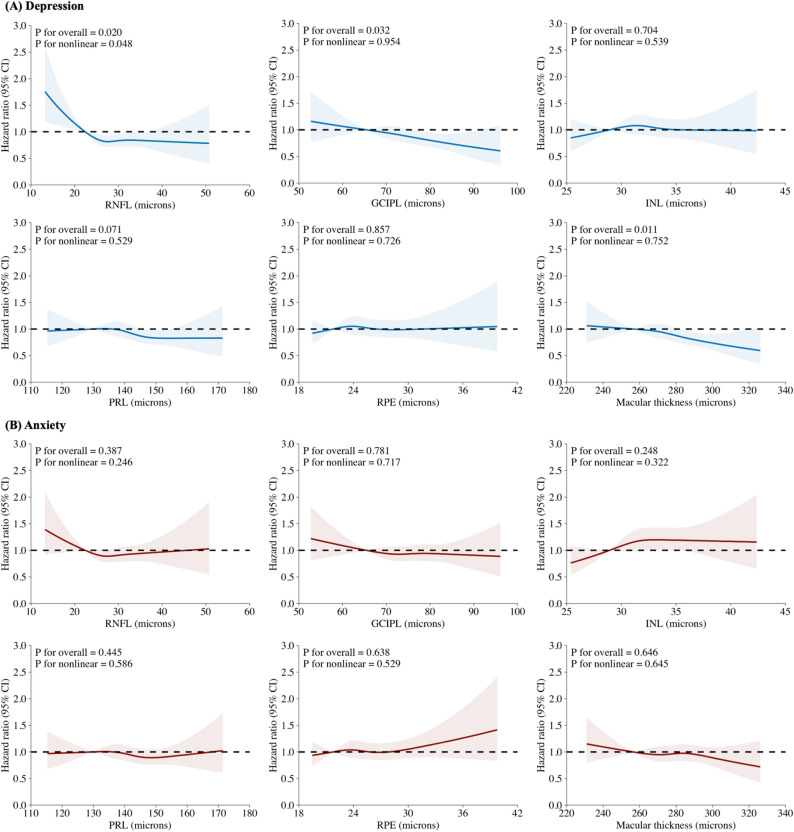
Restricted cubic spline plots for the association between retinal features with incident depression and anxiety disorders

The RCS analysis confirmed a linear association in GCIPL (*P* for nonlinearity = 0.95) and macular thickness (*P* for nonlinearity = 0.75) with incident depression risk. We also observed a non-linear association between RNFL and incident depression (*P* for nonlinearity = 0.048). On the other hand, we observed no linear or non-linear dependency of incident anxiety risk on these retinal features in the RCS models (Fig. 2).

### Subgroup and sensitivity analysis

**Fig. 3:**
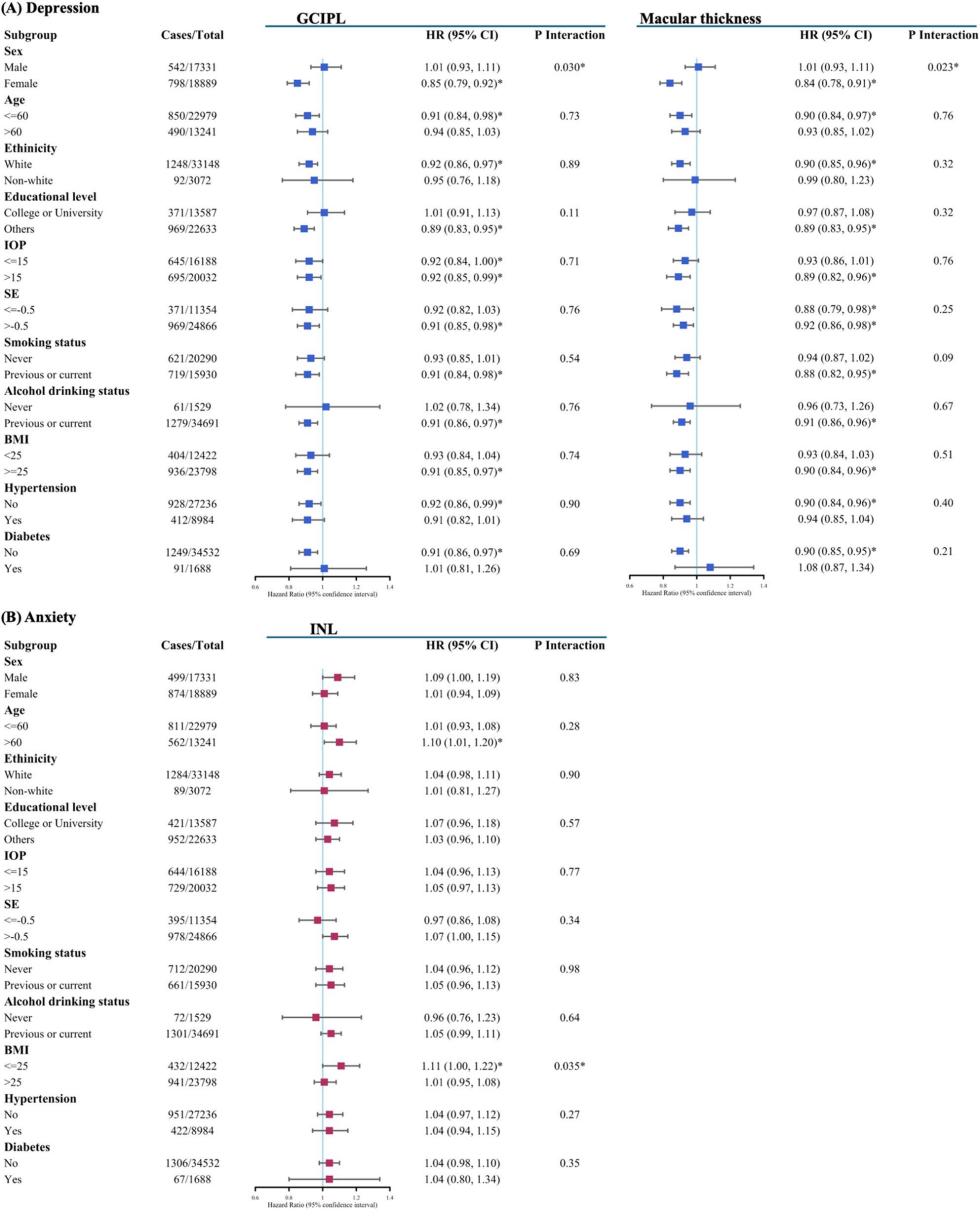
Subgroup analysis for GCIPL, INL, and macular thickness

Subgroup analysis revealed interaction effects of association in the sex and BMI groups (Fig. 3, Additional file 1: Tables S3-4, Figures S3-4). When stratifying individuals by sex, the associations between GCIPL and macular thickness with incident depression were more striking in female participants (all *P* for interaction < 0.05). Specifically, per SD increase in GCIPL was related to a 15% reduction in the incidence of depression (HR:0.85, 95%CI: 0.79–0.92, *P* < 0.001), while per SD increase in macular thickness was linked to 16% reduction (HR:0.84, 95%CI: 0.78–0.91, *P* < 0.001) among females. Furthermore, the association between INL and incident anxiety was modified by BMI group (*P* for interaction = 0.035). In participants with a BMI less than 25, per SD increase in INL was associated with 11% increased risk of incident anxiety (HR:1.11, 95%CI:1.00-1.22, *P* = 0.049).

The robustness of our findings was further supported by a series of sensitivity analyses. Consistent results were observed after excluding participants diagnosed with diseases within the first four years of follow-up, as well as those with self-reported ocular diseases or high refractive errors, those with specific retinal conditions, or those with neurological disorders (Additional file 1: Tables S5-S8). Additional analyses that adjusted for physical activity, sleep duration, and the intake of mood-affecting medications did not change the magnitude or direction of the identified associations (Additional file 1: Tables S9-10).

## Discussion

In the analysis of 36,220 participants from the UK Biobank who underwent retinal optical coherence tomography imaging, we found specific retinal features associated with incident depression and anxiety disorders. First, per SD reduction in both GCIPL and total macular thickness was associated with 8% and 9% increased risk of depression. Compared to those in the lowest quartile of GCIPL and macular thickness, individuals in the highest quartile had a 17% and 24% reduced risk of depression. In addition, the inverse associations between GCIPL, macular thickness and the risk of depression were more pronounced among women. Second, while the overall association of INL thickness with incident anxiety was not significant in the fully adjusted model, an effect modification by BMI was observed, with a more pronounced association in individuals with normal or low BMI. Our findings suggest that retinal morphology is associated with the risk of developing mental illnesses.

In this study, we observed a cumulative incidence of 3.7% for depression and 3.8% for anxiety disorders within the UK Biobank cohort over a median follow-up of 12.5 years, aligning with previous UK Biobank studies [27]. Our observed depression incidence was slightly lower than that reported in a 12-year Danish nationwide cohort study [28], which found an incidence of 5.6% in men and 10.2% in women. The observed incidence of anxiety disorders was comparable to a 10-year longitudinal study, which reported a 4.3% incidence [29]. 

Previous studies have identified several retinal features correlated with MRI-derived parameters during the process of neurodegeneration in both neurological and psychiatric diseases [30–32]. Depression has been linked to altered connectivity and function, as well as structural changes in the brain [7]. In patients diagnosed with major depressive disorders (MDD), retinal neurovascular alterations have been observed, including decreased RNFL and ganglion complex cell (GCC) thickness and optic nerve head volume [33]. Kalenderoglu et al. [34] reported decreased ganglion cell layer (GCL) and inner plexiform layer (IPL) volumes in 100 MDD patients, which comprised 50 patients with recurrent episodes and 50 with first episodes. They found a negative correlation between both GCL and IPL volumes with disease severity and duration. Notably, RNFL did not significantly differ between the MDD and control groups, suggesting that RNFL may be the latest layer affected during the degeneration process associated with MDD. Furthermore, Friedel et al. [20] revealed an inverse association between GCIPL and depressive symptoms in a sample of 31 MDD patients, suggesting that GCIPL may act as an indicator of cumulative neurodegenerative processes related to depression. However, these studies were cross-sectional designs, which limit the assessment of the prognostic value of retinal features. Our longitudinal analysis demonstrates that individuals with a thinner GCIPL and macular thickness exhibit a higher risk of developing depression later in life, especially in women. This suggests that GCIPL and macular thinning are associated with an increased risk of developing depression.

The mechanism underlying the association of thinning of GCIPL with the development of depression is not well understood. Accumulating evidence suggested that patients with depression may exhibit abnormalities of the prefrontal cortex in the brain [35, 36]. GCIPL was formed by somas and dendrites of the retinal ganglion cells (RGCs), which interact with neurons in the retina and the brain [37]. Experiment studies have revealed a mood-regulating circuit that connects intrinsically photosensitive retinal ganglion cells to the prefrontal cortical region, which may play a role in the pathophysiology of mood disorders [38]. 

Research on retinal structural changes in anxiety disorders is scarce. One cross-sectional study by Ozisik et al. found that patients with newly diagnosed generalized anxiety disorder had a significantly thinner GCIPL compared to age- and gender-matched healthy controls [39]. On the contrary, Acan et al. [40] reported no significant alteration in retinal morphology or vascular density, but found increased subfoveal choroidal thickness and a reduced choroidal vascular index in patients with anxiety disorders as assessed by OCT-angiography. The discrepancies between these studies may be attributed to variations in the OCT devices used and the heterogeneity of clinical characteristics and disease stages of the studied populations.

Notably, our findings indicate a marginal association between INL and risk of anxiety in the crude model. The association between thicker INL and incident anxiety was more evident in participants with non-obese BMI. The INL comprises three types of neuronal cells (horizontal, bipolar, and amacrine cells) and Müller glial cells, which play roles in neurotransmission and homeostasis in the retina [41]. Thickening of INL may result from inflammatory processes or disruptions in retinal fluid homoeostasis [42], potentially reflecting neuroplastic adaptations to chronic stress or anxiety that altered emotional processing. Numerous studies have reported associations between increased INL thickness and early stages of glaucoma [43]. Besides, thickening of INL can be indicative of greater IOP fluctuation and subsequent visual field progression in early stages of glaucomatous eyes [44, 45]. Given that low body size and anxiety disorders have been identified as potential precursors to glaucoma [46, 47], future studies are warranted to explore the INL thickening and the development of anxiety disorders, particularly in participants with normal or low body size.

The strengths of this study included a large sample size with objectively measured OCT imaging data and an average follow-up duration of more than 10 years to investigate the influence of retinal features on the incidence of depression and anxiety. However, there are some limitations for this study. First, although we excluded incident cases occurring within the first four years, reverse causality cannot be completely ruled out, given the insidious prodromal phases of depression and anxiety disorders. Second, depression and anxiety cases identified solely on ICD-10 codes obtained from inpatient hospital records may lead to an underestimation of the true incidence. Individuals with atypical clinical symptoms or milder forms of these disorders might not have been captured. Our findings may predominantly reflect associations with more severe manifestations of these conditions. Third, the UK Biobank cohort is primarily composed of individuals of White ethnicity. Moreover, potential selection bias may stem from excluding participants with suboptimal retinal image quality, who were generally older, non-White, and less healthy. Therefore, our findings may not be fully generalizable to other populations. Fourth, the subgroup analysis and observed interaction effects regarding sex and BMI should be considered exploratory due to multiple testing, and the results should be interpreted with caution. Finally, our study only includes retinal measurements at a single time point (baseline recruitment). Future studies incorporating longitudinal measurements of retinal features are required to explore the dynamic changes in retinal morphology and to elucidate causal relationships during the development of depression and anxiety.

## Conclusions

In conclusion, our study provided evidence for the association between retinal morphological features and risk of depression, though no independent association was found with incident anxiety disorders. Future studies are needed to validate the longitudinal relationship between retinal morphology and the development of mental disorders.

## Supplementary Information

Below is the link to the electronic supplementary material.

Supplementary Material 1: Additional File 1: Tables S1-S10, Figures S1-S4.

## Data Availability

The data used in this study is available in the UK Biobank database under the application number 91320. Further details can be found on the UK Biobank website (https://www.ukbiobank.ac.uk).

## Abbreviations

BMI: body mass index
FDR: false discovery rate
GCC: ganglion complex cell
GCIPL: ganglion cell-inner plexiform layer
HR: hazard ratio
IOP: intraocular pressure
INL: inner nuclear layer
MDD: major depressive disorders
MRI: Magnetic resonance imaging
OCT: optical coherence tomography
PRL: photoreceptor layer
RCS: restricted cubic spline
RGC: retinal ganglion cell
RNFL: Retinal nerve fibre layer
RPE: retinal pigment epithelium
SD: standard deviation
SE: spherical equivalent
TABS: Topcon Advanced Boundary Segmentation
TDI: Townsend deprivation index
