# Supplementary material for: Associations between retinal morphological features and risk of depression and anxiety disorders
Source: BMC Med. 2026 Jun 5;24:433. doi: 10.1186/s12916-026-04969-8 (PMC13464321; doi:10.1186/s12916-026-04969-8)
Supplement: Supplementary file 1 [file 12916_2026_4969_MOESM1_ESM.docx]

**Associations between Retinal Morphological Features and Risk of Depression and Anxiety Disorders**

**Additional file 1**

**Supplementary Table Legends**

**Table S1.** Baseline characteristics of included and excluded participants.

**Table S2.** Baseline characteristics of participants by incident depression and anxiety disorders status.

**Table S3.** Stratified analyses of the associations between retinal morphological features and the risk of incident depression.

**Table S4.** Stratified analyses of the associations between retinal morphological features and the risk of incident anxiety.

**Table S5.** Associations between retinal features and incident depression and anxiety, excluding incidence in the first four years.

**Table S6.** Associations between retinal features and incident depression and anxiety, excluding participants with baseline self-reported eye diseases or high refractive errors (> ±6 diopters).

**Table S7.** Associations between retinal features and incident depression and anxiety, excluding participants with specific retinal conditions (glaucoma, AMD, or DR).

**Table S8.** Associations between retinal features and incident depression and anxiety, excluding participants with baseline neurological disorders.

**Table S9.** Associations between retinal features and incident depression and anxiety, with further adjustment for physical activity, sleep duration, and mood-affecting medications.

**Table S10.** Number of participants had regular mood-affecting medications at baseline recruitment.

**Supplementary Figure Legends**

**Figure S1.** Participants flow charts.

**Figure S2**. The histogram of OCT parameters.

**Figure S3.** Associations between quartiles of GCIPL and macular thickness and incident depression, stratified by sex.

**Figure S4.** Associations between quartiles of INL and incident anxiety, stratified by BMI.

**Table S1.** Baseline characteristics of included and excluded participants.

|  | Included (N=36,220) | Excluded (N=32,234) | *P* value |
| --- | --- | --- | --- |
| **Demographics** |  |  |  |
| Age, mean (SD), years | 55.90 (8.22) | 57.75 (7.95) | **<0.001** |
| Sex, male, No. (%) | 17331 (47.9%） | 13929 (43.2%) | **<0.001** |
| TDI, mean (SD) | -1.14 (2.92) | -0.87 (3.08) | **<0.001** |
| Ethnicity, White, No. (%) | 33148 (91.5%) | 28395 (88.7%) | **<0.001** |
| Education level, college, No.(%) | 13587 (37.5%) | 10451 (33.4%) | **<0.001** |
| Smoking status, No. (%) |  |  | **<0.001** |
| Never | 20290 (56.0%) | 17479 (55.0%) |  |
| Previous | 12652 (34.9%) | 10951 (34.5%) |  |
| Current | 3278 (9.1%) | 3349 (10.5%) |  |
| Alcohol drinking status, No. (%) |  |  | **<0.001** |
| Never | 1529 (4.2%) | 1831 (5.7%) |  |
| Previous | 1116 (3.1%) | 1341 (4.2%) |  |
| Current | 33575 (92.7%) | 28778 (90.1%) |  |
| BMI, mean (SD) | 27.20 (4.64) | 27.57 (4.94) | **<0.001** |
| Spherical equivalent, mean (SD), D | -0.26 (2.33) | -0.45 (2.98) | **<0.001** |
| Intraocular pressure, mean (SD), mmHg | 15.44 (2.95) | 16.30 (4.34) | **<0.001** |
| Hypertension, No. (%) | 8984 (24.8%) | 9408 (29.2%) | **<0.001** |
| Diabetes, No. (%) | 1688 (4.7%) | 2134 (6.6%) | **<0.001** |

Data were presented as mean (standard deviation) for continuous variables and No.(%) for categorical variables.

**Table S2.** Baseline characteristics of participants by incident depression and anxiety disorders status.

|  | **Incident depression disorders** | | |  | **Incident anxiety disorders** | | |
| --- | --- | --- | --- | --- | --- | --- | --- |
|  | No (N=34880) | Yes (N=1340) | *P* value |  | No (N=34847) | Yes (N=1373) | *P* value |
| **Demographics** |  |  |  |  |  |  |  |
| Age, mean (SD), years | 55.91 (8.22) | 55.80 (8.25) | 0.63 |  | 55.88 (8.22) | 56.44 (8.43) | **0.016** |
| Sex, male, No. (%) | 16789 (48.1%) | 542 (40.4%) | **<0.001** |  | 16832 (48.3%) | 499 (36.3%) | **<0.001** |
| TDI, mean (SD) | -1.16 (2.91) | -0.60 (3.09) | **<0.001** |  | -1.14 (2.92) | -0.93 (2.98) | **0.008** |
| Ethnicity, No. (%) |  |  | **0.031** |  |  |  | **0.007** |
| White | 31900 (91.5%) | 1248 (93.1%) |  |  | 31864 (91.4%) | 1284 (93.5%) |  |
| Non-white | 2980 (8.5%) | 92 (6.9%) |  |  | 2983 (8.6%) | 89 (6.5%) |  |
| Education level |  |  | **<0.001** |  |  |  | **<0.001** |
| College or university | 13216 (37.9%) | 371 (27.7%) |  |  | 13166 (37.8%) | 421 (30.7%) |  |
| Others | 21664 (62.1%) | 969 (72.3%) |  |  | 21681 (62.2%) | 952 (69.3%) |  |
| Smoking status, No. (%) |  |  | **<0.001** |  |  |  | **<0.001** |
| Never | 19669 (56.4%) | 621 (46.3%) |  |  | 19578 (56.2%) | 712 (51.9%) |  |
| Previous | 12161 (34.9%) | 491 (36.6%) |  |  | 12174 (34.9%) | 478 (34.8%) |  |
| Current | 3050 (8.7%) | 228 (17.0%) |  |  | 3095 (8.9%) | 183 (13.3%) |  |
| Alcohol drinking status |  |  | <0.001 |  |  |  | **<0.001** |
| Never | 1468 (4.2%) | 61 (4.6%) |  |  | 1457 (4.2%) | 72 (5.2%) |  |
| Previous | 1032 (3.0%) | 84 (6.3%) |  |  | 1043 (3.0%) | 73 (5.3%) |  |
| Current | 32380 (92.8%) | 1195 (89.2%) |  |  | 32347 (92.8%) | 1228 (89.4%) |  |
| BMI, mean (SD) | 27.17 (4.60) | 28.19 (5.42) | **<0.001** |  | 27.18 (4.61) | 27.77 (5.24) | **<0.001** |
| Spherical equivalent, mean (SD), D | -0.26 (2.33) | -0.08 (2.29) | **0.004** |  | -0.27 (2.33) | -0.02 (2.27) | **<0.001** |
| Intraocular pressure, mean (SD), mmHg | 15.45 (2.94) | 15.19 (3.06) | **0.002** |  | 15.45 (2.95) | 15.32 (3.03) | 0.12 |
| Hypertension, No. (%) | 8572 (24.6%) | 412 (30.7%) | **<0.001** |  | 8562 (24.6%) | 422 (30.7%) | **<0.001** |
| Diabetes, No. (%) | 1597 (4.6%) | 91 (6.8%) | **<0.001** |  | 1306 (3.8%) | 67 (4.0%) | 0.69 |
| **Retinal markers** |  |  |  |  |  |  |  |
| RNFL, mean (SD), microns | 28.59 (4.20) | 28.33 (4.25) | **0.023** |  | 28.59 (4.20) | 28.49 (4.27) | 0.42 |
| GC-IPL, mean (SD), microns | 74.57 (5.55) | 74.25 (5.45) | **0.040** |  | 74.56 (5.54) | 74.54 (5.56) | 0.92 |
| INL, mean (SD), microns | 32.54 (2.30) | 32.56 (2.23) | 0.72 |  | 32.54 (2.30) | 32.64 (2.24) | 0.11 |
| PRL, mean (SD), microns | 142.22 (7.64) | 141.51 (7.60) | **<0.001** |  | 142.21 (7.64) | 141.79 (7.60) | **0.045** |
| RPE, mean (SD), microns | 25.28 (2.78) | 25.23 (2.77) | 0.54 |  | 25.28 (2.78) | 25.24 (2.79) | 0.64 |
| Macular thickness, mean (SD), microns | 277.94 (13.20) | 276.66 (12.88) | **<0.001** |  | 277.90 (13.20) | 277.48 (12.95) | 0.24 |

Data were presented as mean (standard deviation) for continuous variables and No.(%) for categorical variables.

**Table S3.** Stratified analyses of the associations between retinal morphological features and the risk of incident depression.

|  |  | **RNFL** | | | **GCIPL** | | | **INL** | | |
| --- | --- | --- | --- | --- | --- | --- | --- | --- | --- | --- |
|  | **Events** | HR (95% CI) | *P* value | *P* Interaction | HR (95% CI) | *P* value | *P* Interaction | HR (95% CI) | *P* value | *P* Interaction |
| **Subgroup** |  |  |  |  |  |  |  |  |  |  |
| **Sex** |  |  |  | 0.38 |  |  | **0.030** |  |  | 0.07 |
| Male | 542/17331 | 0.97 (0.89, 1.06) | 0.53 |  | 1.01 (0.93, 1.11) | 0.75 |  | 1.09 (0.99, 1.18) | 0.07 |  |
| Female | 798/18889 | 0.93 (0.87, 1.01) | 0.07 |  | 0.85 (0.79, 0.92) | **<0.001** |  | 0.92 (0.85, 0.99) | **0.037** |  |
| **Age** |  |  |  | 0.49 |  |  | 0.73 |  |  | 0.31 |
| <=60 | 850/22979 | 0.96 (0.90, 1.04) | 0.31 |  | 0.91 (0.84, 0.98) | **0.011** |  | 0.96 (0.89, 1.03) | 0.29 |  |
| >60 | 490/13241 | 0.93 (0.85, 1.02) | 0.11 |  | 0.94 (0.85, 1.03) | 0.18 |  | 1.04 (0.95, 1.14) | 0.38 |  |
| **Ethinicity** |  |  |  | 0.58 |  |  | 0.89 |  |  | 0.24 |
| White | 1248/33148 | 0.95 (0.89, 1.00) | 0.07 |  | 0.92 (0.86, 0.97) | **0.004** |  | 0.98 (0.92, 1.04) | 0.44 |  |
| Non-white | 92/3072 | 0.98 (0.78, 1.22) | 0.82 |  | 0.95 (0.76, 1.18) | 0.64 |  | 1.14 (0.91, 1.42) | 0.26 |  |
| **Educational level** |  |  |  | 0.90 |  |  | 0.11 |  |  | 0.33 |
| College | 371/13587 | 0.97 (0.87, 1.08) | 0.58 |  | 1.01 (0.91, 1.13) | 0.84 |  | 1.03 (0.92, 1.15) | 0.57 |  |
| Others | 969/22633 | 0.94 (0.88, 1.01) | 0.08 |  | 0.89 (0.83, 0.95) | **<0.001** |  | 0.97 (0.91, 1.04) | 0.42 |  |
| **IOP** |  |  |  | 0.50 |  |  | 0.71 |  |  | 0.57 |
| <=15 | 645/16188 | 0.97 (0.89, 1.05) | 0.44 |  | 0.92 (0.84, 1.00) | **0.045** |  | 1.02 (0.94, 1.10) | 0.71 |  |
| >15 | 695/20032 | 0.93 (0.86, 1.01) | 0.09 |  | 0.92 (0.85, 0.99) | **0.029** |  | 0.96 (0.89, 1.04) | 0.36 |  |
| **SE** |  |  |  | 0.64 |  |  | 0.76 |  |  | 0.81 |
| <=-0.5 | 371/11354 | 0.98 (0.89, 1.08) | 0.71 |  | 0.92 (0.82, 1.03) | 0.15 |  | 0.98 (0.88, 1.11) | 0.79 |  |
| >-0.5 | 969/24866 | 0.94 (0.87, 1.00) | 0.06 |  | 0.91 (0.85, 0.98) | **0.008** |  | 0.99 (0.92, 1.06) | 0.71 |  |
| **Smoking status** |  |  |  | 0.14 |  |  | 0.54 |  |  | 0.55 |
| Non-smoker | 621/20290 | 1.01 (0.92, 1.08) | 0.89 |  | 0.93 (0.85, 1.01) | 0.08 |  | 1.00 (0.92, 1.08) | 0.93 |  |
| Smoker | 719/15930 | 0.91 (0.84, 0.99) | **0.021** |  | 0.91 (0.84, 0.98) | **0.014** |  | 0.98 (0.91, 1.06) | 0.63 |  |
| **Alcohol drinking status** | |  |  | 0.15 |  |  | 0.76 |  |  | 0.82 |
| Never | 61/1529 | 1.06 (0.82, 1.37) | 0.66 |  | 1.02 (0.78, 1.34) | 0.87 |  | 1.06 (0.82, 1.39) | 0.65 |  |
| Previous or current | 1279/34691 | 0.95 (0.89, 1.00) | 0.06 |  | 0.91 (0.86, 0.97) | **0.003** |  | 0.98 (0.93, 1.04) | 0.6 |  |
| **BMI** |  |  |  | 0.48 |  |  | 0.74 |  |  | 0.20 |
| <=25 | 404/12422 | 0.94 (0.85, 1.04) | 0.24 |  | 0.93 (0.84, 1.04) | 0.19 |  | 1.04 (0.93, 1.15) | 0.52 |  |
| >25 | 936/23798 | 0.95 (0.89, 1.02) | 0.16 |  | 0.91 (0.85, 0.97) | **0.007** |  | 0.97 (0.90, 1.04) | 0.35 |  |
| **Hypertension** |  |  |  | 0.28 |  |  | 0.90 |  |  | 0.85 |
| No | 928/27236 | 0.97 (0.91, 1.04) | 0.38 |  | 0.92 (0.86, 0.99) | **0.02** |  | 0.99 (0.92, 1.06) | 0.69 |  |
| Yes | 412/8984 | 0.91 (0.82, 1.00) | 0.06 |  | 0.91 (0.82, 1.01) | 0.08 |  | 1.00 (0.90, 1.11) | 0.95 |  |
| **Diabetes** |  |  |  | **0.014** |  |  | 0.69 |  |  | 0.47 |
| No | 1249/34532 | 0.93 (0.88, 0.99) | **0.024** |  | 0.91 (0.86, 0.97) | **0.002** |  | 0.99 (0.93, 1.05) | 0.75 |  |
| Yes | 91/1688 | 1.16 (0.95, 1.43) | 0.14 |  | 1.01 (0.81, 1.26) | 0.91 |  | 0.95 (0.76, 1.19) | 0.65 |  |
|  |  | **PRL** | | | **RPE** | | | **Macular thickness** | | |
|  | **Events** | HR (95% CI) | *P* value | *P* Interaction | HR (95% CI) | *P* value | *P* Interaction | HR (95% CI) | *P* value | *P* Interaction |
| **Subgroup** |  |  |  |  |  |  |  |  |  |  |
| **Sex** |  |  |  | 0.20 |  |  | 0.49 |  |  | **0.023** |
| Male | 542/17331 | 1.00 (0.92, 1.09) | 0.96 |  | 1.02 (0.94, 1.11) | 0.69 |  | 1.01 (0.93, 1.11) | 0.78 |  |
| Female | 798/18889 | 0.89 (0.83, 0.96) | **0.002** |  | 0.97 (0.90, 1.05) | 0.45 |  | 0.84 (0.78, 0.91) | **<0.001** |  |
| **Age** |  |  |  | 0.76 |  |  | 0.14 |  |  | 0.76 |
| <=60 | 850/22979 | 0.93 (0.87, 1.00) | **0.045** |  | 1.01 (0.95, 1.08) | 0.69 |  | 0.90 (0.84, 0.97) | **0.006** |  |
| >60 | 490/13241 | 0.95 (0.86, 1.04) | 0.26 |  | 0.93 (0.84, 1.03) | 0.16 |  | 0.93 (0.85, 1.02) | 0.12 |  |
| **Ethinicity** |  |  |  | 0.25 |  |  | 0.38 |  |  | 0.32 |
| White | 1248/33148 | 0.93 (0.87, 0.98) | **0.009** |  | 0.98 (0.93, 1.04) | 0.54 |  | 0.90 (0.85, 0.96) | **<0.001** |  |
| Non-white | 92/3072 | 1.00 (0.82, 1.22) | 0.99 |  | 1.12 (0.94, 1.32) | 0.2 |  | 0.99 (0.80, 1.23) | 0.94 |  |
| **Educational level** |  |  |  | 0.76 |  |  | 0.67 |  |  | 0.32 |
| College | 371/13587 | 0.95 (0.86, 1.06) | 0.40 |  | 0.97 (0.87, 1.08) | 0.54 |  | 0.97 (0.87, 1.08) | 0.62 |  |
| Others | 969/22633 | 0.93 (0.87, 0.99) | **0.023** |  | 1.00 (0.94, 1.06) | 0.95 |  | 0.89 (0.83, 0.95) | **<0.001** |  |
| **IOP** |  |  |  | 0.80 |  |  | 0.56 |  |  | 0.76 |
| <=15 | 645/16188 | 0.95 (0.88, 1.03) | 0.24 |  | 0.98 (0.90, 1.06) | 0.55 |  | 0.93 (0.86, 1.01) | 0.09 |  |
| >15 | 695/20032 | 0.92 (0.85, 0.99) | **0.032** |  | 1.00 (0.93, 1.08) | 0.92 |  | 0.89 (0.82, 0.96) | **0.004** |  |
| **SE** |  |  |  | **0.049** |  |  | 0.88 |  |  | 0.25 |
| <=-0.5 | 371/11354 | 0.86 (0.77, 0.96) | **0.006** |  | 1.00 (0.89, 1.13) | 0.98 |  | 0.88 (0.79, 0.98) | **0.019** |  |
| >-0.5 | 969/24866 | 0.96 (0.90, 1.03) | 0.24 |  | 0.99 (0.93, 1.05) | 0.66 |  | 0.92 (0.86, 0.98) | **0.015** |  |
| **Smoking status** |  |  |  | 0.12 |  |  | 0.65 |  |  | 0.09 |
| Non-smoker | 621/20290 | 0.96 (0.88, 1.04) | 0.30 |  | 0.98 (0.91, 1.07) | 0.67 |  | 0.94 (0.87, 1.02) | 0.16 |  |
| Smoker | 719/15930 | 0.91 (0.85, 0.99) | **0.023** |  | 1.00 (0.93, 1.08) | 0.99 |  | 0.88 (0.82, 0.95) | **0.001** |  |
| **Alcohol drinking status** | |  |  | 0.73 |  |  | 0.31 |  |  | 0.67 |
| Never | 61/1529 | 0.88 (0.67, 1.15) | 0.33 |  | 1.15 (0.91, 1.46) | 0.25 |  | 0.96 (0.73, 1.26) | 0.79 |  |
| Previous or current | 1279/34691 | 0.94 (0.88, 0.99) | **0.023** |  | 0.99 (0.93, 1.04) | 0.62 |  | 0.91 (0.86, 0.96) | **0.001** |  |
| **BMI** |  |  |  | 0.39 |  |  | 0.62 |  |  | 0.51 |
| <=25 | 404/12422 | 0.95 (0.86, 1.06) | 0.37 |  | 0.96 (0.86, 1.06) | 0.39 |  | 0.93 (0.84, 1.03) | 0.19 |  |
| >25 | 936/23798 | 0.92 (0.86, 0.99) | **0.021** |  | 1.01 (0.94, 1.08) | 0.83 |  | 0.90 (0.84, 0.96) | **0.002** |  |
| **Hypertension** |  |  |  | 0.06 |  |  | 0.70 |  |  | 0.40 |
| No | 928/27236 | 0.90 (0.84, 0.96) | **0.003** |  | 1.00 (0.94, 1.07) | 0.98 |  | 0.90 (0.84, 0.96) | **0.002** |  |
| Yes | 412/8984 | 1.01 (0.92, 1.12) | 0.78 |  | 0.96 (0.87, 1.07) | 0.50 |  | 0.94 (0.85, 1.04) | 0.23 |  |
| **Diabetes** |  |  |  | 0.44 |  |  | **0.046** |  |  | 0.21 |
| No | 1249/34532 | 0.92 (0.87, 0.98) | **0.008** |  | 1.00 (0.95, 1.06) | 0.88 |  | 0.90 (0.85, 0.95) | **<0.001** |  |
| Yes | 91/1688 | 1.05 (0.85, 1.29) | 0.66 |  | 0.80 (0.63, 1.02) | 0.07 |  | 1.08 (0.87, 1.34) | 0.48 |  |

Abbreviations: RNFL, retinal nerve fiber layer; GCIPL, ganglion cell-inner plexiform layer; INL, inner nuclear layer; PRL, photoreceptor layer; RPE, retinal pigment epithelium; BMI, body mass index; TDI, Townsend deprivation index. IOP, intraocular pressure; SE, spherical equivalent.

All models were adjusted for age, sex, TDI, ethnicity, education level, smoking status, alcohol drinking status, BMI, SE, IOP, hypertension, and diabetes.

**Table S4.** Stratified analyses of the associations between retinal morphological features and the risk of incident anxiety.

|  |  | **RNFL** | | | **GCIPL** | | | **INL** | | |
| --- | --- | --- | --- | --- | --- | --- | --- | --- | --- | --- |
|  | **Events** | HR (95% CI) | *P* value | *P* Interaction | HR (95% CI) | *P* value | *P* Interaction | HR (95% CI) | *P* value | *P* Interaction |
| **Subgroup** |  |  |  |  |  |  |  |  |  |  |
| **Sex** |  |  |  | 0.25 |  |  | 0.34 |  |  | 0.83 |
| Male | 499/17331 | 1.00 (0.91, 1.10) | 0.99 |  | 1.05 (0.96, 1.15) | 0.32 |  | 1.09 (1.00, 1.19) | 0.06 |  |
| Female | 874/18889 | 0.98 (0.92, 1.05) | 0.63 |  | 0.94 (0.87, 1.01) | 0.09 |  | 1.01 (0.94, 1.09) | 0.72 |  |
| **Age** |  |  |  | 0.68 |  |  | 0.82 |  |  | 0.28 |
| <=60 | 811/22979 | 0.98 (0.91, 1.05) | 0.52 |  | 0.97 (0.90, 1.05) | 0.45 |  | 1.01 (0.93, 1.08) | 0.85 |  |
| >60 | 562/13241 | 1.00 (0.92, 1.09) | 0.92 |  | 1.01 (0.93, 1.10) | 0.79 |  | 1.10 (1.01, 1.20) | **0.027** |  |
| **Ethinicity** |  |  |  | 0.60 |  |  | 0.42 |  |  | 0.90 |
| White | 1284/33148 | 0.99 (0.94, 1.05) | 0.73 |  | 0.98 (0.92, 1.04) | 0.48 |  | 1.04 (0.98, 1.11) | 0.15 |  |
| Non-white | 89/3072 | 0.94 (0.74, 1.19) | 0.59 |  | 1.03 (0.83, 1.29) | 0.77 |  | 1.01 (0.81, 1.27) | 0.92 |  |
| **Educational level** |  |  |  | 0.28 |  |  | 0.64 |  |  | 0.57 |
| College | 421/13587 | 0.96 (0.87, 1.05) | 0.37 |  | 0.97 (0.87, 1.08) | 0.56 |  | 1.07 (0.96, 1.18) | 0.23 |  |
| Others | 952/22633 | 1.00 (0.94, 1.07) | 0.89 |  | 0.99 (0.92, 1.06) | 0.72 |  | 1.03 (0.96, 1.10) | 0.38 |  |
| **IOP** |  |  |  | 0.73 |  |  | 0.81 |  |  | 0.77 |
| <=15 | 644/16188 | 0.99 (0.91, 1.08) | 0.85 |  | 0.99 (0.91, 1.08) | 0.84 |  | 1.04 (0.96, 1.13) | 0.39 |  |
| >15 | 729/20032 | 0.99 (0.92, 1.06) | 0.73 |  | 0.98 (0.90, 1.05) | 0.52 |  | 1.05 (0.97, 1.13) | 0.26 |  |
| **SE** |  |  |  | 0.58 |  |  | 0.38 |  |  | 0.34 |
| <=-0.5 | 395/11354 | 1.02 (0.93, 1.12) | 0.65 |  | 0.93 (0.83, 1.04) | 0.19 |  | 0.97 (0.86, 1.08) | 0.55 |  |
| >-0.5 | 978/24866 | 0.97 (0.91, 1.04) | 0.45 |  | 1.00 (0.94, 1.07) | 0.92 |  | 1.07 (1.00, 1.15) | **0.036** |  |
| **Smoking status** |  |  |  | 0.36 |  |  | 0.31 |  |  | 0.98 |
| Non-smoker | 712/20290 | 0.96 (0.89, 1.03) | 0.27 |  | 0.95 (0.87, 1.02) | 0.17 |  | 1.04 (0.96, 1.12) | 0.37 |  |
| Smoker | 661/15930 | 1.02 (0.94, 1.11) | 0.59 |  | 1.02 (0.94, 1.11) | 0.59 |  | 1.05 (0.96, 1.13) | 0.28 |  |
| **Alcohol drinking status** | |  |  | 0.66 |  |  | 0.98 |  |  | 0.64 |
| Never | 72/1529 | 1.02 (0.80, 1.30) | 0.88 |  | 0.95 (0.74, 1.22) | 0.67 |  | 0.96 (0.76, 1.23) | 0.77 |  |
| Previous or current | 1301/34691 | 0.99 (0.93, 1.05) | 0.67 |  | 0.98 (0.93, 1.04) | 0.61 |  | 1.05 (0.99, 1.11) | 0.13 |  |
| **BMI** |  |  |  | 0.08 |  |  | 0.47 |  |  | **0.035** |
| <=25 | 432/12422 | 0.94 (0.85, 1.04) | 0.25 |  | 1.00 (0.90, 1.10) | 0.93 |  | 1.11 (1.00, 1.22) | **0.049** |  |
| >25 | 941/23798 | 1.01 (0.94, 1.08) | 0.8 |  | 0.98 (0.91, 1.05) | 0.49 |  | 1.01 (0.95, 1.08) | 0.72 |  |
| **Hypertension** |  |  |  | 0.35 |  |  | 0.44 |  |  | 0.27 |
| No | 951/27236 | 0.98 (0.92, 1.05) | 0.61 |  | 0.98 (0.91, 1.05) | 0.54 |  | 1.04 (0.97, 1.12) | 0.22 |  |
| Yes | 422/8984 | 1.00 (0.90, 1.10) | 0.95 |  | 0.99 (0.89, 1.10) | 0.85 |  | 1.04 (0.94, 1.15) | 0.42 |  |
| **Diabetes** |  |  |  | 0.60 |  |  | 0.21 |  |  | 0.35 |
| No | 1306/34532 | 0.99 (0.94, 1.05) | 0.85 |  | 0.98 (0.93, 1.04) | 0.6 |  | 1.04 (0.98, 1.10) | 0.17 |  |
| Yes | 67/1688 | 0.88 (0.69, 1.13) | 0.32 |  | 0.92 (0.72, 1.19) | 0.54 |  | 1.04 (0.80, 1.34) | 0.79 |  |
|  |  | **PRL** | | | **RPE** | | | **Macular thickness** | | |
|  | **Events** | HR (95% CI) | *P* value | *P* Interaction | HR (95% CI) | *P* value | *P* Interaction | HR (95% CI) | *P* value | *P* Interaction |
| **Subgroup** |  |  |  |  |  |  |  |  |  |  |
| **Sex** |  |  |  | 0.99 |  |  | 0.80 |  |  | 0.42 |
| Male | 499/17331 | 0.99 (0.90, 1.08) | 0.75 |  | 1.02 (0.93, 1.11) | 0.71 |  | 1.03 (0.94, 1.12) | 0.57 |  |
| Female | 874/18889 | 0.95 (0.89, 1.02) | 0.17 |  | 1.02 (0.95, 1.09) | 0.64 |  | 0.94 (0.88, 1.01) | 0.11 |  |
| **Age** |  |  |  | 0.18 |  |  | 0.25 |  |  | 0.23 |
| <=60 | 811/22979 | 0.94 (0.87, 1.01) | 0.07 |  | 1.03 (0.96, 1.10) | 0.37 |  | 0.94 (0.88, 1.02) | 0.12 |  |
| >60 | 562/13241 | 1.03 (0.94, 1.12) | 0.57 |  | 0.98 (0.89, 1.08) | 0.7 |  | 1.04 (0.95, 1.13) | 0.4 |  |
| **Ethinicity** |  |  |  | 0.84 |  |  | 0.72 |  |  | 0.76 |
| White | 1284/33148 | 0.96 (0.91, 1.02) | 0.22 |  | 1.02 (0.96, 1.08) | 0.48 |  | 0.97 (0.92, 1.03) | 0.38 |  |
| Non-white | 89/3072 | 0.97 (0.79, 1.18) | 0.74 |  | 0.99 (0.83, 1.18) | 0.89 |  | 0.98 (0.79, 1.21) | 0.82 |  |
| **Educational level** |  |  |  | 0.73 |  |  | 0.71 |  |  | 0.51 |
| College | 421/13587 | 0.95 (0.86, 1.05) | 0.28 |  | 1.01 (0.91, 1.12) | 0.82 |  | 0.95 (0.86, 1.05) | 0.33 |  |
| Others | 952/22633 | 0.97 (0.91, 1.04) | 0.42 |  | 1.02 (0.95, 1.09) | 0.6 |  | 0.99 (0.92, 1.05) | 0.67 |  |
| **IOP** |  |  |  | **0.030** |  |  | 0.24 |  |  | 0.27 |
| <=15 | 644/16188 | 0.91 (0.84, 0.98) | **0.018** |  | 1.05 (0.98, 1.14) | 0.19 |  | 0.94 (0.87, 1.02) | 0.17 |  |
| >15 | 729/20032 | 1.02 (0.94, 1.10) | 0.65 |  | 0.98 (0.91, 1.06) | 0.66 |  | 1.00 (0.93, 1.08) | 0.94 |  |
| **SE** |  |  |  | 0.39 |  |  | 0.89 |  |  | 0.39 |
| <=-0.5 | 395/11354 | 0.91 (0.82, 1.01) | 0.08 |  | 1.01 (0.90, 1.13) | 0.86 |  | 0.92 (0.83, 1.03) | 0.14 |  |
| >-0.5 | 978/24866 | 0.98 (0.92, 1.05) | 0.63 |  | 1.02 (0.96, 1.09) | 0.51 |  | 1.00 (0.93, 1.06) | 0.91 |  |
| **Smoking status** |  |  |  | 0.32 |  |  | 0.38 |  |  | 0.89 |
| Non-smoker | 712/20290 | 0.99 (0.91, 1.06) | 0.71 |  | 1.04 (0.96, 1.12) | 0.32 |  | 0.96 (0.89, 1.04) | 0.33 |  |
| Smoker | 661/15930 | 0.95 (0.87, 1.03) | 0.17 |  | 0.99 (0.92, 1.08) | 0.89 |  | 0.99 (0.91, 1.07) | 0.83 |  |
| **Alcohol drinking status** | |  |  | 0.52 |  |  | 0.76 |  |  | 0.67 |
| Never | 72/1529 | 1.02 (0.80, 1.30) | 0.88 |  | 0.95 (0.75, 1.19) | 0.65 |  | 0.99 (0.78, 1.26) | 0.93 |  |
| Previous or current | 1301/34691 | 0.96 (0.91, 1.02) | 0.17 |  | 1.02 (0.97, 1.08) | 0.45 |  | 0.97 (0.92, 1.03) | 0.37 |  |
| **BMI** |  |  |  | 0.23 |  |  | 0.62 |  |  | 0.42 |
| <=25 | 432/12422 | 1.00 (0.90, 1.10) | 0.92 |  | 0.98 (0.89, 1.08) | 0.65 |  | 0.99 (0.90, 1.10) | 0.9 |  |
| >25 | 941/23798 | 0.95 (0.89, 1.02) | 0.13 |  | 1.04 (0.97, 1.11) | 0.27 |  | 0.97 (0.90, 1.03) | 0.31 |  |
| **Hypertension** |  |  |  | 0.76 |  |  | 0.27 |  |  | 0.69 |
| No | 951/27236 | 0.96 (0.90, 1.03) | 0.25 |  | 1.03 (0.96, 1.10) | 0.41 |  | 0.97 (0.91, 1.04) | 0.38 |  |
| Yes | 422/8984 | 0.98 (0.88, 1.08) | 0.63 |  | 0.99 (0.89, 1.10) | 0.85 |  | 0.99 (0.89, 1.09) | 0.81 |  |
| **Diabetes** |  |  |  | 0.49 |  |  | 0.72 |  |  | 0.20 |
| No | 1306/34532 | 0.96 (0.91, 1.02) | 0.21 |  | 1.02 (0.96, 1.08) | 0.55 |  | 0.98 (0.92, 1.04) | 0.44 |  |
| Yes | 67/1688 | 0.98 (0.76, 1.26) | 0.87 |  | 1.01 (0.77, 1.31) | 0.95 |  | 0.92 (0.72, 1.19) | 0.53 |  |

Abbreviations: RNFL, retinal nerve fiber layer; GCIPL, ganglion cell-inner plexiform layer; INL, inner nuclear layer; PRL, photoreceptor layer; RPE, retinal pigment epithelium; BMI, body mass index; TDI, Townsend deprivation index. IOP, intraocular pressure; SE, spherical equivalent.

All models were adjusted for age, sex, TDI, ethnicity, education level, smoking status, alcohol drinking status, BMI, SE, IOP, hypertension, and diabetes.

**Table S5.** Associations between retinal features and incident depression and anxiety, excluding incidence in the first four years

|  |  | Model1 |  |  | Model 2 |  |
| --- | --- | --- | --- | --- | --- | --- |
|  | Events | Per SD increase HR (95%CI) | *P* value |  | Per SD increase HR (95%CI) | *P* value |
| **Depression** | 972/35852 |  |  |  |  |  |
| RNFL |  | 0.92 (0.87, 0.99) | 0.018* |  | 0.94 (0.88, 1.01) | 0.09 |
| GCIPL |  | 0.93 (0.87, 0.99) | 0.017* |  | 0.90 (0.84, 0.96) | **0.002*** |
| INL |  | 1.03 (0.97, 1.10) | 0.35 |  | 1.01 (0.94, 1.08) | 0.88 |
| PRL |  | 0.92 (0.87, 0.98) | 0.015* |  | 0.93 (0.87, 0.99) | 0.023* |
| RPE |  | 1.01 (0.95, 1.08) | 0.59 |  | 1.02 (0.95, 1.08) | 0.64 |
| Macular thickness |  | 0.91 (0.85, 0.97) | **0.003*** |  | 0.90 (0.84, 0.96) | **0.002*** |
| **Anxiety** | 1060/35907 |  |  |  |  |  |
| RNFL |  | 0.98 (0.92, 1.04) | 0.47 |  | 1.00 (0.94, 1.07) | 0.99 |
| GCIPL |  | 1.00 (0.94, 1.06) | 0.96 |  | 0.98 (0.92, 1.04) | 0.49 |
| INL |  | 1.06 (1.00, 1.13) | 0.042 |  | 1.04 (0.98, 1.11) | 0.21 |
| PRL |  | 0.96 (0.90, 1.02) | 0.16 |  | 0.96 (0.90, 1.02) | 0.15 |
| RPE |  | 1.02 (0.96, 1.08) | 0.61 |  | 1.01 (0.95, 1.08) | 0.71 |
| Macular thickness |  | 0.98 (0.92, 1.04) | 0.47 |  | 0.97 (0.91, 1.04) | 0.37 |

Abbreviations: RNFL, retinal nerve fiber layer; GCIPL, ganglion cell-inner plexiform layer; INL, inner nuclear layer; PRL, photoreceptor layer; RPE, retinal pigment epithelium; BMI, body mass index; TDI, Townsend deprivation index. IOP, intraocular pressure; SE, spherical equivalent.

Model 1 was adjusted for age, sex, TDI, ethnicity, and education level;

Model 2 was adjusted for age, sex, TDI, ethnicity, education level, smoking status, alcohol drinking status, BMI, SE, IOP, hypertension, and diabetes.

* indicate FDR-corrected *P* value <0.1; Bold values indicate FDR-corrected *P* value <0.05.

**Table S6.** Associations between retinal features and incident depression and anxiety, excluding participants with baseline self-reported eye diseases or high refractive errors (> ±6 diopters).

|  |  | Model1 |  |  | Model 2 |  |
| --- | --- | --- | --- | --- | --- | --- |
|  | Events | Per SD increase HR (95%CI) | *P* value |  | Per SD increase HR (95%CI) | *P* value |
| **Depression** | 1173/32538 |  |  |  |  |  |
| RNFL |  | 0.93 (0.87, 0.98) | **0.012*** |  | 0.95 (0.89, 1.01) | 0.09 |
| GCIPL |  | 0.94 (0.89, 1.00) | 0.041* |  | 0.92 (0.86, 0.98) | **0.006*** |
| INL |  | 1.01 (0.96, 1.08) | 0.14 |  | 0.99 (0.93, 1.05) | 0.70 |
| PRL |  | 0.92 (0.87, 0.97) | 0.022* |  | 0.94 (0.88, 1.00) | 0.037 |
| RPE |  | 0.98 (0.93, 1.04) | 0.60 |  | 0.98 (0.92, 1.04) | 0.52 |
| Macular thickness |  | 0.92 (0.86, 0.97) | **0.004*** |  | 0.91 (0.86, 0.97) | **0.003*** |
| **Anxiety** | 1190/32538 |  |  |  |  |  |
| RNFL |  | 0.96 (0.90, 1.02) | 0.16 |  | 0.98 (0.92, 1.04) | 0.51 |
| GCIPL |  | 1.02 (0.96, 1.08) | 0.49 |  | 1.00 (0.94, 1.06) | 0.93 |
| INL |  | 1.08 (1.02, 1.14) | **0.009*** |  | 1.05 (0.99, 1.12) | 0.09 |
| PRL |  | 0.97 (0.92, 1.03) | 0.34 |  | 0.97 (0.91, 1.03) | 0.27 |
| RPE |  | 1.01 (0.95, 1.07) | 0.78 |  | 1.00 (0.94, 1.06) | 0.97 |
| Macular thickness |  | 0.99 (0.94, 1.05) | 0.80 |  | 0.98 (0.92, 1.04) | 0.55 |

Abbreviations: RNFL, retinal nerve fiber layer; GCIPL, ganglion cell-inner plexiform layer; INL, inner nuclear layer; PRL, photoreceptor layer; RPE, retinal pigment epithelium; BMI, body mass index; TDI, Townsend deprivation index. IOP, intraocular pressure; SE, spherical equivalent.

Model 1 was adjusted for age, sex, TDI, ethnicity, and education level;

Model 2 was adjusted for age, sex, TDI, ethnicity, education level, smoking status, alcohol drinking status, BMI, SE, IOP, hypertension, and diabetes.

* indicate FDR-corrected *P* value <0.1; Bold values indicate FDR-corrected *P* value <0.05.

Self-reported eye disorders included cataract (n=1258), glaucoma (n=399), age-related macular degeneration (n=288), diabetic retinopathy (n=286), vision loss caused by trauma or injury (n=151), and other serious eye conditions (n=468).

**Table S7.** Associations between retinal features and incident depression and anxiety, excluding participants with specific retinal conditions (glaucoma, AMD, or DR).

|  | Model1 (exclude baseline DR) | | |  | Model 2 (exclude baseline AMD) | | |  | Model 3 (exclude baseline glaucoma) | | |
| --- | --- | --- | --- | --- | --- | --- | --- | --- | --- | --- | --- |
|  | Events | Per SD increase HR (95%CI) | *P* value |  | Events | Per SD increase HR (95%CI) | *P* value |  | Events | Per SD increase HR (95%CI) | *P* value |
| **Depression** | 1323/35934 | |  |  | 1320/35932 | |  |  | 1315/35821 | |  |
| RNFL |  | 0.95 (0.90, 1.00) | 0.07 |  |  | 0.95 (0.90, 1.01) | 0.09 |  |  | 0.95 (0.90, 1.01) | 0.10 |
| GCIPL |  | 0.92 (0.87, 0.97) | **0.004*** |  |  | 0.92 (0.87, 0.98) | **0.005*** |  |  | 0.91 (0.86, 0.97) | **0.003*** |
| INL |  | 0.99 (0.94, 1.05) | 0.79 |  |  | 0.99 (0.94, 1.05) | 0.81 |  |  | 0.98 (0.93, 1.04) | 0.57 |
| PRL |  | 0.93 (0.88, 0.98) | **0.012*** |  |  | 0.93 (0.88, 0.99) | 0.018* |  |  | 0.93 (0.88, 0.98) | **0.011*** |
| RPE |  | 0.99 (0.94, 1.05) | 0.79 |  |  | 0.99 (0.94, 1.05) | 0.80 |  |  | 0.99 (0.93, 1.04) | 0.62 |
| Macular thickness |  | 0.91 (0.86, 0.96) | **<0.001*** |  |  | 0.91 (0.86, 0.97) | **0.002*** |  |  | 0.91 (0.86, 0.96) | **<0.001*** |
| **Anxiety** | 1356/35934 | |  |  | 1358/35932 | |  |  | 1342/35821 | |  |
| RNFL |  | 0.99 (0.94, 1.05) | 0.77 |  |  | 0.99 (0.93, 1.04) | 0.64 |  |  | 1.00 (0.95, 1.06) | 0.97 |
| GCIPL |  | 0.99 (0.93, 1.04) | 0.62 |  |  | 0.98 (0.93, 1.04) | 0.56 |  |  | 0.99 (0.94, 1.05) | 0.84 |
| INL |  | 1.05 (0.99, 1.11) | 0.13 |  |  | 1.04 (0.98, 1.10) | 0.15 |  |  | 1.04 (0.98, 1.10) | 0.15 |
| PRL |  | 0.96 (0.91, 1.02) | 0.20 |  |  | 0.97 (0.91, 1.02) | 0.24 |  |  | 0.96 (0.91, 1.02) | 0.18 |
| RPE |  | 1.02 (0.96, 1.07) | 0.57 |  |  | 1.02 (0.96, 1.07) | 0.59 |  |  | 1.01 (0.96, 1.07) | 0.70 |
| Macular thickness |  | 0.98 (0.92, 1.03) | 0.43 |  |  | 0.98 (0.92, 1.03) | 0.41 |  |  | 0.98 (0.93, 1.04) | 0.55 |

Abbreviations: AMD, age-related macular degeneration; DR, diabetic retinopathy; RNFL, retinal nerve fiber layer; GCIPL, ganglion cell-inner plexiform layer; INL, inner nuclear layer; PRL, photoreceptor layer; RPE, retinal pigment epithelium; BMI, body mass index; TDI, Townsend deprivation index. IOP, intraocular pressure; SE, spherical equivalent.

All models were adjusted for age, sex, TDI, ethnicity, education levels, smoking status, alcohol drinking status, BMI, SE, IOP, hypertension, and diabetes.

* indicate FDR-corrected *P* value<0.1; Bold value indicate FDR-corrected *P* value<0.05.

**Table S8.** Associations between retinal features and incident depression and anxiety, excluding participants with baseline neurological disorders.

|  |  | Model1 |  |  | Model 2 |  |
| --- | --- | --- | --- | --- | --- | --- |
|  | Events | Per SD increase HR (95%CI) | *P* value |  | Per SD increase HR (95%CI) | *P* value |
| **Depression** | 1304/35701 |  |  |  |  |  |
| RNFL |  | 0.93 (0.88, 0.99) | **0.017*** |  | 0.95 (0.90, 1.01) | 0.08 |
| GCIPL |  | 0.94 (0.88, 0.99) | **0.018*** |  | 0.92 (0.87, 0.97) | **0.004*** |
| INL |  | 1.01 (0.95, 1.06) | 0.85 |  | 0.99 (0.93, 1.05) | 0.66 |
| PRL |  | 0.93 (0.88, 0.98) | **0.011*** |  | 0.94 (0.89, 0.99) | 0.029 |
| RPE |  | 0.99 (0.94, 1.05) | 0.76 |  | 0.99 (0.94, 1.05) | 0.78 |
| Macular thickness |  | 0.91 (0.86, 0.97) | **0.001*** |  | 0.91 (0.86, 0.97) | **0.002*** |
| **Anxiety** |  |  |  |  |  |  |
| RNFL | 1340/35701 | 0.96 (0.91, 1.01) | 0.15 |  | 0.98 (0.93, 1.04) | 0.49 |
| GCIPL |  | 1.00 (0.95, 1.06) | 0.96 |  | 0.98 (0.93, 1.04) | 0.51 |
| INL |  | 1.07 (1.01, 1.13) | **0.018*** |  | 1.05 (0.99, 1.11) | 0.13 |
| PRL |  | 0.97 (0.92, 1.02) | 0.28 |  | 0.97 (0.92, 1.02) | 0.26 |
| RPE |  | 1.02 (0.97, 1.08) | 0.40 |  | 1.02 (0.96, 1.08) | 0.51 |
| Macular thickness |  | 0.98 (0.93, 1.04) | 0.51 |  | 0.97 (0.92, 1.03) | 0.37 |

Abbreviations: RNFL, retinal nerve fiber layer; GCIPL, ganglion cell-inner plexiform layer; INL, inner nuclear layer; PRL, photoreceptor layer; RPE, retinal pigment epithelium; BMI, body mass index; TDI, Townsend deprivation index. IOP, intraocular pressure; SE, spherical equivalent.

Model 1 was adjusted for age, sex, TDI, ethnicity, and education level;

Model 2 was adjusted for age, sex, TDI, ethnicity, education level, smoking status, alcohol drinking status, BMI, SE, IOP, hypertension, and diabetes.

* indicate FDR-corrected *P* value<0.1; Bold value indicate FDR-corrected *P* value<0.05.

**Table S9.** Associations between retinal features and incident depression and anxiety, with further adjustment for physical activity, sleep duration, and mood-affecting medications.

|  | Events | Per SD increase HR (95%CI) | *P* value |
| --- | --- | --- | --- |
| **Depression** | 1340/36220 |  |  |
| RNFL |  | 0.95 (0.90, 1.01) | 0.09 |
| GCIPL |  | 0.92 (0.87, 0.98) | **0.005*** |
| INL |  | 0.98 (0.93, 1.04) | 0.59 |
| PRL |  | 0.95 (0.90, 1.00) | 0.06 |
| RPE |  | 0.99 (0.93, 1.04) | 0.63 |
| Macular thickness |  | 0.92 (0.87, 0.97) | **0.004*** |
| **Anxiety** | 1373/36220 |  |  |
| RNFL |  | 0.99 (0.94, 1.05) | 0.71 |
| GCIPL |  | 0.99 (0.93, 1.04) | 0.61 |
| INL |  | 1.04 (0.98, 1.10) | 0.18 |
| PRL |  | 0.97 (0.92, 1.03) | 0.32 |
| RPE |  | 1.01 (0.96, 1.07) | 0.61 |
| Macular thickness |  | 0.98 (0.93, 1.04) | 0.49 |

Abbreviations: RNFL, retinal nerve fiber layer; GCIPL, ganglion cell-inner plexiform layer; INL, inner nuclear layer; PRL, photoreceptor layer; RPE, retinal pigment epithelium; BMI, body mass index; TDI, Townsend deprivation index. IOP, intraocular pressure; SE, spherical equivalent.

Models were adjusted for age, sex, TDI, ethnicity, education level, smoking status, alcohol drinking status, BMI, physical activity, sleep duration, SE, IOP, hypertension, diabetes, and intake of mood-affecting medications.

* indicate FDR-corrected *P* value<0.1; Bold value indicate FDR-corrected *P* value<0.05.

**Table S10.** Number of participants had regular mood-affecting medications at baseline recruitment

| Mood-affecting medication | Frequency |
| --- | --- |
| Antidepressants | 756 (2.1%) |
| Antipsychotics | 98 (0.3%) |
| Anxiolytics | 125 (3.5%) |
| Mood stabilizers | 27 (0.1%) |
| Beta-blockers | 1967 (5.4%) |
| Corticosteroids | 254 (0.7%) |
| Anticonvulsants | 450 (1.2%) |
| Total | 3307 (9.1%) |

Participants may have been prescribed more than one mood-affecting medication.

**Figure S1.** Participants flow chart.

IOP, intraocular pressure; TDI, Townsend deprivation index; BMI, body mass index; logMAR, logarithm of the minimum angle of resolution;

**Figure S2.** The histogram of OCT parameters.

**Figure S3.** Associations between quartiles of GCIPL and macular thickness and incident depression, stratified by sex.

**Figure S4.** Associations between quartiles of INL and incident anxiety, stratified by BMI.
